# Hemorrhagic transformation after endovascular treatment: Baseline infarct volume is a better predictor than infarct growth rate

**DOI:** 10.1093/esj/23969873251357151

**Published:** 2026-01-01

**Authors:** Mathilde Méot, Fanny Munsch, Bertrand Lapergue, Maeva Kyheng, Igor Sibon, David Planes, Emilien Micard, Bailiang Chen, Jean-Marc Olivot, Grégoire Boulouis, Alain Viguier, Thomas Tourdias, Gaultier Marnat

**Affiliations:** Neuroradiology Department, Bordeaux University Hospital, Bordeaux, France; Institut de Bio-Imagerie IBIO, Bordeaux University, Bordeaux, France; Neurology Department, Foch Hospital, Suresnes, France; Biostatistics Department, Lille University Hospital, Lille, France; Neurology Department, Bordeaux University Hospital, Bordeaux, France; Neuroradiology Department, Bordeaux University Hospital, Bordeaux, France; Inserm CIC-IT U1433, CHRU Nancy, Nancy, France; Inserm CIC-IT U1433, CHRU Nancy, Nancy, France; Neurology Department, Toulouse University Hospital, Toulouse, France; CIC 1436, Toulouse University, France; Neuroradiology Department, Tours University Hospital, Tours, France; Neurology Department, Toulouse University Hospital, Toulouse, France; Neuroradiology Department, Bordeaux University Hospital, Bordeaux, France; Institut de Bio-Imagerie IBIO, Bordeaux University, Bordeaux, France; Neuroradiology Department, Bordeaux University Hospital, Bordeaux, France

**Keywords:** Ischemic stroke, infarct growth rate, intracranial hemorrhage, endovascular treatment%

## Abstract

**Background and objectives:**

Hemorrhagic transformation (HT) remains an important issue following ischemic stroke. Efforts have been made to identify predictors of HT, especially imaging features. Among them, the infarct growth rate (IGR) remains underexplored. We investigated the influence of IGR on the risk of subsequent HT in the setting of large vessel occlusion stroke (LVOS) intended for endovascular treatment (EVT) and compared IGR to baseline infarct volume as predictors of HT.

**Methods:**

We conducted a secondary analysis of two merged prospectively collected databases (FRAME 2017–2019 and ETIS 2015–2021). Patients presenting with anterior circulation LVOS, a witnessed symptoms onset, baseline MRI within 24 h after symptoms onset and available day 1 imaging (MRI or CT) were included. Posterior circulation LVOS, medium and distal vessel occlusions of the anterior circulation, tandem occlusions and unknown time of stroke onset were excluded. The primary endpoint was the occurrence of any HT detected on day 1 imaging. Secondary endpoint was the occurrence of parenchymal hematoma (defined as PH1 or PH2). Associations between the IGR and the occurrence of any HT and parenchymal hematoma within 24-h after mechanical thrombectomy were assessed using univariable and multivariable logistic regression models.

**Results:**

We included 775 patients (mean age 70.5 years (SD 15.1)). The median of IGR was 8.7 ml per hour (IQR 2.8–24.2). A faster IGR was independently associated with a higher risk of any HT (adjusted OR 1.35; 95% CI 1.16–1.57 per one log unit increase). A faster IGR was also associated with an increased risk of parenchymal hemorrhage in univariate analysis (OR 1.35; 95% CI 1.15–1.58), but the association did not remain significant in multivariable analysis including all the other predictors of parenchymal hemorrhage (adjusted OR 1.16 (95% CI 0.96–1.40) per one log unit increase). ROC analyses revealed that baseline infarct volume significantly better predicted any HT and PH occurrence than the IGR (*p* = 0.019 and *p* = 0.029 respectively).

**Conclusion:**

In patients presenting with anterior circulation LVOS and treated with EVT, the IGR was significantly associated with an increased risk of HT. However, the baseline infarct volume was a stronger predictor of HT than IGR.

## Graphical Abstract

**Graphical abstract:**
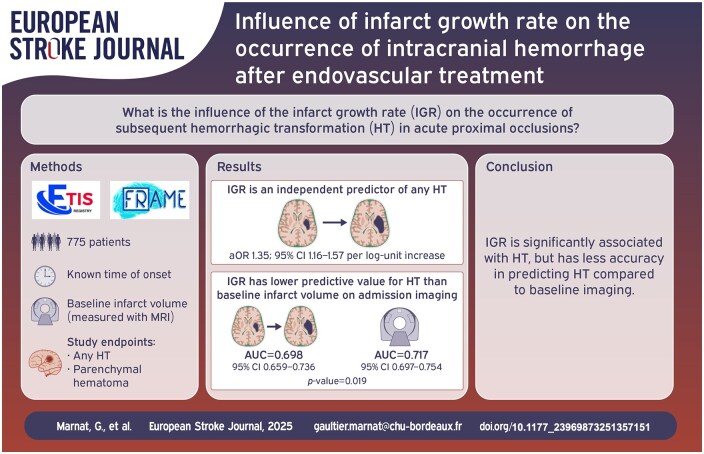

## Introduction

Hemorrhagic Transformation (HT) is a frequent complication after acute ischemic stroke, in particular in large vessel occlusion stroke (LVOS) after emergent reperfusion therapies including intravenous thrombolysis (IVT) and/or endovascular treatment (EVT).^1^ HT remains a key issue at the early phase of ischemic stroke negatively influencing clinical outcome through direct (irreversible parenchymal injuries, local mass effect, edema, intracranial hypertension) and indirect consequences (extended hospital stay, delayed introduction of secondary prevention anticoagulant treatments).^2,3^

Previous studies identified predictors of HT in the acute phase of ischemic stroke. Most important were sex, diabetes, hypercholesterolemia, hypertension, initial NIHSS, atrial fibrillation and baseline ASPECTS.^3–5^ Mechanisms leading to HT following ischemic stroke are complex and intricated, involving blood-brain barrier (BBB) disorders, inflammation, vascular dysfunction and coagulation disorders.^6,7^

Among imaging derived information, infarct growth rate (IGR), defined as the infarct volume on initial imaging divided by the time from stroke onset, might be a valuable feature. However, despite recently pointed, its independent role on outcomes remains poorly investigated to date.^8–13^ Longer time from stroke onset and larger ischemic core on baseline imaging have already separately been found associated with an increased risk of HT.^5,14,15^ IGR may aggregate these two information into a single metric that might allow an individualized evaluation of the HT risk. But whether IGR could provide a stronger prognosis information than the well-established prediction of the baseline infarct volume alone remains unknown. Among the existing literature, there are few indirect evidence that the pattern of IGR may influence HT occurrence.^11,16–19^ However, the impact of the pattern of IGR, especially within the earliest hours after stroke onset, on the subsequent likelihood of HT remains to be elucidated. Such an understanding could not only help the understanding of stroke pathophysiology, but also inform clinical decisions for a more precise and personalized management of high-risk patients. It could also imply the development of prevention strategies to reduce the risk of HT.

Analyzing the clinical and imaging data from a large cohort of ischemic stroke, we sought to identify patterns of IGR associated with an increased risk of early HT. Our study aimed to investigate the influence of IGR on the risk of subsequent HT in comparison with baseline infarct volume in the setting of LVOS intended for mechanical thrombectomy.

## Methods

### Study design and data sources

We conducted a secondary analysis using data from two prospective studies: *French Acute multimodal Imaging Study to Select Patients for Mechanical Thrombectomy* (FRAME; NCT03045146) and *Endovascular Treatment in Ischemic Stroke* (ETIS; NCT03776877). The same population has already been used to highlight the archetype dynamic evolution of IGR in time and space^16^ but without focus on the possible association with HT. Our analysis was conducted according to the reporting guideline for observational studies.

Briefly, ETIS is an ongoing, prospective, multicentric observational registry collecting clinical, procedural and imaging data from consecutive patients receiving endovascular treatment for LVOS in the anterior circulation. Twenty-one French comprehensive stroke centers were involved in this registry at the time of this study. For the purpose of this work, we collected imaging and clinical data of patients included from 1st January 2015 to 31st December 2021. Details about data collection and materials have already been published.^5,20^

FRAME was a prospective, multicenter cohort study involving 2 French comprehensive stroke centers. Recruited patients presented with LVOS, from January 2017 to January 2019, explored with acute imaging including perfusion-weighted imaging on arrival and treated with EVT until 6 h after onset.^21^

This research was approved by the local ethics committee at each center. In both registries (ETIS and FRAME), each participant signed a written informed consent form without financial compensation.

For the current analysis, we included patients presenting with: (1) a witnessed symptoms onset, (2) baseline MRI including Diffusion Weighted Imaging (DWI) within 24 h from symptoms onset, (3) an anterior circulation LVOS (M1 and/or intracranial internal carotid artery (ICA) occlusion) intended for EVT, and (4) an available day 1 imaging (MRI or CT). For consistency with daily practice, patients undergoing an early recanalization on the initial angiographic run (before any mechanical thrombectomy pass) or experiencing a failure to access the occlusion site during EVT were included in the study population along with all subjects undergoing mechanical thrombectomy, either successfully or not, as per definition of the intention for EVT. Exclusion criteria were as follow: patients without available baseline MRI and/or day 1 follow-up (MRI or CT), posterior circulation LVOS, medium size vessel occlusions of the anterior circulation (M2, A1), tandem occlusions and unknown time of stroke onset.

### Imaging data

All included patients underwent MRI at admission and MRI or CT at day 1. All imaging and angiographic variables were collected by trained senior neuroradiologists. On admission MRI, the following imaging variables were collected: (1) the volume of infarct core (defined as the volume of apparent diffusion coefficient < 620.10^−6^ mm^2^/s on diffusion weighted imaging), (2) the arterial occlusion site (intracranial ICA or the M1 segment of the middle cerebral artery).

For the ETIS patients, the collateral status was evaluated on digital subtraction angiography obtained during EVT, using the American Society of Interventional and Therapeutic Neuroradiology Collateral Grading System (ASITN CS) defined classification from 0 to 4. Scores of 0, 1, and 2 were considered as poor collateral and 3 and 4 as good collateral status.^22–24^ In the FRAME cohort, an angiographic assessment of the collateral status was not available. The hypoperfusion index ratio (HIR) defined as the volume of tissue with *T*max >10 s divided by the volume of tissue with *T*max >6 s was used as a proxy of the collateral status. We dichotomized as good collateral status (HIR < 0.4) and poor collateral status (HIR > 0.4).^21^

The IGR was calculated dividing the core volume on baseline DWI (baseline infarct volume) by the time between symptoms onset and imaging. The value was presented in milliliter per hour (ml/h).

Day 1 imaging were performed at 24 ± 12 h from symptoms onset. HT was assessed on day 1 imaging according to the European Collaborative Acute Stroke Study II definition.^25,26^ Hemorrhagic infarction 1 (HI1) was defined as small petechiae along the margins of the infarct and hemorrhagic infarction 2 (HI2) as confluent petechiae within the infarcted area but no space-occupying effect. Parenchymal hematoma 1 (PH1) was defined as blood clots in 30% or less of the infarcted area with some slight space occupying effect and parenchymal hematoma 2 (PH2) as blood clots in more than 30% of the infarcted area with substantial space-occupying. HT evaluation was performed blindly from the value of IGR. In the FRAME derived subpopulation, HT was centrally assessed by a core lab. In patients from the ETIS registry, HT was locally assessed in participating centers by trained senior neuroradiologists, also blindly from the IGR value.

The recanalization status after mechanical thrombectomy was assessed using the modified Thrombolysis In Cerebral Infarction (mTICI) score. A favorable recanalization was defined as final mTICI of 2b, 2c, or 3.

### Study endpoints

The primary endpoint was the occurrence of any HT detected on day 1 imaging. Secondary endpoint was the occurrence of a parenchymal hematoma (defined as PH1 or PH2).

### Statistical analysis

Categorical variables were expressed as frequencies and percentages. Continuous variables were expressed as mean (standard deviation) or median (interquartile range) for non-normal distribution. Normality of distributions was assessed graphically and by using the Shapiro-Wilk test.

Associations between the IGR and the occurrence of any HT and parenchymal hematoma within 24-h after mechanical thrombectomy were assessed using univariable and multivariable logistic regression models after apply a log-transformation of IGR given the high skewness of its distribution. We further examined the log-linearity assumption using restricted cubic spline functions and result is expressed as odds ratio (OR) calculated per one log unit increase in IGR. We assessed the independent contribution of IGR to predict any-hemorrhage and parenchymal-hemorrhage by including into multivariable logistic regression model all baseline characteristics independently associated with the studied hemorrhage outcome and the IVT as forced variable. These baseline characteristics were found using firstly univariate analyses with Student *t* test (or Mann-Whitney *U* test in case of non-normal distribution) for quantitative characteristics and chi-square test for categorical characteristics) and secondly by using a backward-stepwise selection procedure (including all baseline characteristics associate with *p* < 0.20) using a removal criteria of *p* > 0.05. Before developing the multivariable logistic model, the absence of collinearity between the candidate variables was checked by calculating the variance inflation factors (VIF) and we examined the log-linearity assumption for all quantitative characteristics irrespective of previous univariate analyses. A secondary analysis was conducted including only patients with successful recanalization at the end of the endovascular procedure (defined as final mTICI 2b, 2c, or 3). We assessed the heterogeneity in the association between IGR and HT outcomes according to Day-1 imaging modality (MRI or CT).

The baseline volume and IGR could not be included into the same model being obviously strongly collinear. To compare their predictive strengths, we included separately IGR or baseline volume in the multivariable models aiming at predicting the occurrence of any HT and PH, and we compared their area under the Receiver Operating Characteristic (ROC) curves (AUCs).

To avoid case deletion in analyses due to missing data in baseline characteristics and outcomes, univariate and multivariate analyses were performed after handling missing values by multiple imputation using a regression switching approach (chained equations with *m* = 10). Imputation procedure was performed under the missing at random assumption using all variables listed in Table 1 with a predictive mean matching method for quantitative variables and multinomial or binary logistic regression model for categorical variables. Estimates obtained in the different imputed data sets were combined using the Rubin’s rules.

**Table 1.** Baseline characteristics and outcomes in overall population and according to studies (*n* = 775).

Variable	Values	FRAME (*n* = 125)	ETIS (*n* = 650)
Baseline and therapeutic characteristics			
Age	70.5 ± 15.1	70.3 ± 14.4	70.5 ± 15.3
Men	383/775 (49.4)	54/125 (43.2)	329/650 (50.6)
Diabetes	118/767 (15.4)	23/125 (18.4)	95/642 (14.8)
History of hypertension	445/768 (57.9)	74/125 (59.2)	371/643 (57.7)
Hyperlipidemia	233/751 (31.0)	37/125 (29.6)	196/626 (31.3)
History of smoking	148/725 (20.4)	19/125 (15.2)	129/600 (21.5)
History of coronary artery disease	81/598 (13.5)	7/125 (5.6)	74/473 (15.6)
History of prior transient ischemic attack	5/125 (4.0)	5/125 (4.0)	0 (0.0)
History of prior stroke	109/754 (14.5)	13/125 (10.4)	96/629 (15.3)
Drip and ship workflow	333/745 (44.7)	27/124 (21.8)	306/621 (49.3)
Admission NIHSS, median (IQR)	17 (11–21)	18 (13–22)	16 (11–20)
Occlusion site			
M1	616/775 (79.5)	99/125 (79.2)	517/650 (79.5)
ICA	159/775 (20.5)	26/125 (20.8)	133/650 (20.5)
Favorable collateral status	207/709 (29.2)	57/125 (45.6)	150/584 (25.7)
IV thrombolysis	509/774 (65.8)	88/125 (70.4)	421/649 (64.9)
At least one pass of mechanical thrombectomy	729/775 (94.1)	125/125 (100.0)	604/650 (92.9)
General anesthesia	271/764 (35.5)	55/125 (44.0)	216/639 (33.8)
Time from onset to initial MRI, h, median (IQR)	2.2 (1.6–3.1)	2.5 (1.8–3.7)	2.1 (1.6–2.8)
Time from onset to groin puncture, h, median (IQR)	4.2 (3.1–5.4)	3.8 (2.9–4.9)	4.3 (3.2–5.6)
Time from initial MRI to groin puncture, h, median (IQR)	1.4 (1.0–2.6)	1.0 (0.8–1.2)	1.7 (1.1–2.8)
Infarct volume on baseline MRI (ml), median (IQR)	17.6 (7.1–51.7)	20.2 (10.0–67.9)	17.1 (6.1–47.9)
Infarct growth rate (ml/h), median (IQR)	8.7 (2.8–24.2)	9.6 (3.2–28.6)	8.4 (2.7–23.2)
Outcomes			
Any HT on Day 1 imaging	344/677 (50.8)	76/125 (60.8)	268/552 (48.6)
PH on Day 1 imaging	116/677 (17.1)	46/125 (36.8)	70/552 (12.7)
Favorable functional outcome after 3 months (mRS 0–2)	340/705 (48.2)	64/125 (51.2)	276/580 (47.6)
Death at 3 months	123/705 (17.4)	18/125 (14.4)	105/580 (18.1)

ICA: intracranial carotid artery; IQR: interquartile range; IV: intravenous; MRI: magnetic resonance imaging; NIHSS: National Institutes of Health Stroke Scale; PH: parenchymal hemorrhage, SD: standard deviation.

Values expressed as number (percentage) unless otherwise indicated.

All statistical tests were two-sided and *p* < 0.05 was considered statistically significant. Data were analyzed using the SAS software package, release 9.4 (SAS Institute, Cary, NC).

## Results

### Patients characteristics

Between 2015 and 2021, 775 patients with anterior circulation stroke, a known time of symptoms onset and an MRI as baseline imaging type were included in our study. Main patient’s and stroke characteristics are reported in Table 1. The mean age was 70.5 years (SD, 15.1). Patients were men in 49.4% (*n* = 383/775) and more than half had an history of hypertension (*n* = 445/768, 57.9%). Median NIHSS score was 17 (IQR 11–21) and 65.8% of patient received IVT (509/774). The median IGR was of 8.7 ml/h (IQR, 2.8–24.2).

The Day 1 imaging modality was CT in 61.0% (442/725) and MRI in 39.0% (283/725). Among the 677 included patients with available information on HT at day 1, 344 patients (50.8%, 95% CI, 47.0%–54.6%) presented any HT and 116 patients (17.1%, 95% CI, 14.4%–20.2%) had a parenchymal hematoma.

### Association between IGR and any HT

**Figure 1.**
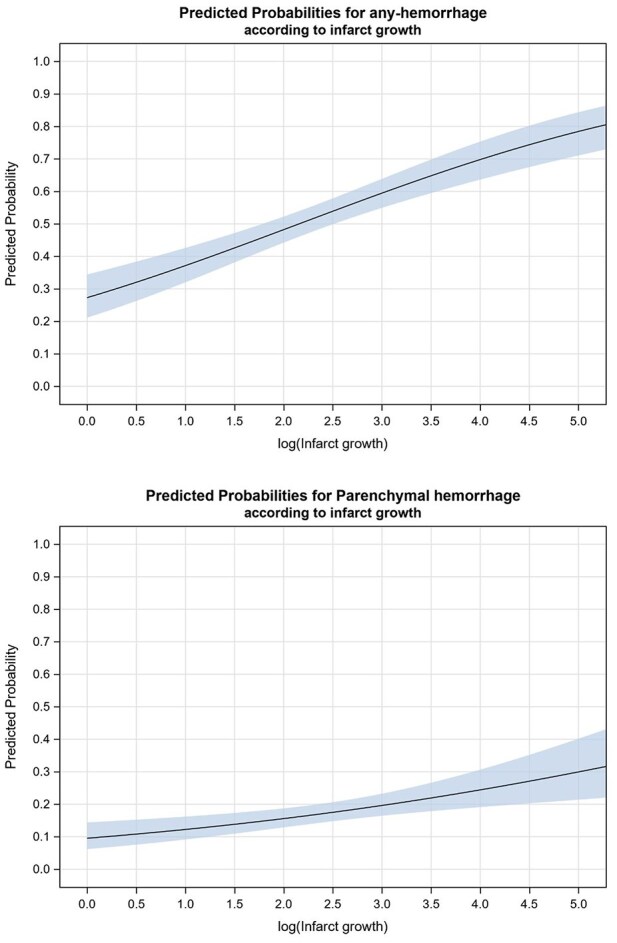
Association of infarct growth with any hemorrhage and parenchymal hemorrhage post-thrombectomy.

As shown in Figure 1 and Table 2, higher IGR values were associated with an increased risk of any HT in univariate analysis, with an OR per one log-increase of 1.54 (95% CI 1.35–1.76). Patients’ characteristics according to occurrence or not of any HT at 24 h are presented in Supplemental Table 1. In multivariable logistic regression analysis including all other predictors of HT (diabetes, drip and ship workflow, occlusion site, IVT, general anesthesia and NIHSS score at baseline (Supplemental Table 2)), IGR remained independently associated with an increased risk of any HT (adjusted OR (aOR) 1.35 (95% CI 1.16–1.57) per one log unit increase).

**Table 2.** Association of infarct growth rate with any HT and parenchymal hemorrhage post-thrombectomy.

Outcomes	Infarct growth rate	Unadjusted OR (95% CI)	*p*-value	Adjusted OR (95% CI)	*p*-value
Any HT					
No	5.1 (1.6–17.2)	1.00 (ref)			
Yes	12.1 (4.3–31.5)	1.54 (1.35–1.76)	<0.001	1.35 (1.16–1.57)^1^	<0.001
PH					
No	7.5 (2.3–22.3)	1.00 (ref)			
Yes	12.9 (4.8–27.6)	1.35 (1.15–1.58)	<0.001	1.16 (0.96–1.40)^2^	0.12

Values are expressed as median (IQR). OR (95% CI) were expressed per one log increase of infarct growth rate.

^1^Model adjusted on IVT and independent factors of any-hemorrhage found in Supplemental Table 2: general anesthesia, drip and ship, diabetes, occlusion site, and NIHSS score at baseline.

^2^Model adjusted on IVT and independent factor of PH found in Supplemental Table 4: diabetes, age, and NIHSS score at baseline. Descriptive parameters and odds ratio were calculated after handling missing values by multiple imputation.

### Association between IGR and parenchymal hematoma

IGR was also associated with an increased risk of parenchymal hematoma in univariate analysis (OR per one log unit increase, 1.35; 95% CI 1.15–1.58). Patients’ characteristics according to occurrence or not of parenchymal hematoma at 24 h are presented in Supplemental Table 3. Despite a trend toward, this association did not remain significant in multivariable analysis including IVT and all the independent predictors of parenchymal hematoma (age, diabetes and NIHSS score at baseline (Supplemental Table 4)) with an aOR of 1.16 (95% CI 0.96–1.40) per one log unit increase (Table 2). Similar results were observed in the secondary analysis focused on patients with final favorable recanalization after EVT (Table 3).

**Table 3.** Association of infarct growth rate with any HT and parenchymal hemorrhage post-thrombectomy in patients with successful recanalization.

Outcomes	Infarct growth rate	Unadjusted OR (95% CI)	*p*-value	Adjusted OR (95% CI)	*p*-value
Any HT					
No	5.0 (1.6–17.1)	1.00 (ref)			
Yes	11.9 (4.1–28.3)	1.50 (1.30–1.73)	<0.001	1.29 (1.09–1.51)^1^	0.002
PH					
No	7.4 (2.2–20.9)	1.00 (ref)			
Yes	12.1 (4.5–26.5)	1.33 (1.12–1.59)	0.001	1.14 (0.93–1.40)^2^	0.21

Values are expressed as median (IQR). OR (95% CI) were expressed per one log increase of infarct growth rate.

^1^Model adjusted on IVT and independent factors of any-hemorrhage found in Supplemental Table 2: general anesthesia, drip and ship, diabetes, occlusion site, and NIHSS score at baseline.

^2^Model adjusted on IVT and independent factor of PH found in Supplemental Table 4: diabetes, age, and NIHSS score at baseline. Descriptive parameters and odds ratio were calculated after handling missing values by multiple imputation.

### Influence of the day 1 imaging modality

We observed no significant heterogeneity in the association between IGR and any HT according to Day-1 imaging modality (*p*-het = 0.42), with an OR (95% CI) of 1.32 (1.11–1.59) for CT and 1.50 (1.17–1.91) for MRI. In contrast, there was significant heterogeneity for PH (*p*-het = 0.034), with an OR (95% CI) of 1.02 (0.82–1.27) for CT and 1.56 (1.12–2.18) for MRI, suggesting a stronger association between IGR and PH when the latter was diagnosed based on MRI on Day 1.

### Comparison of the predictive values of IGR and baseline infarct volume on the occurrence of HT

**Figure 2.**
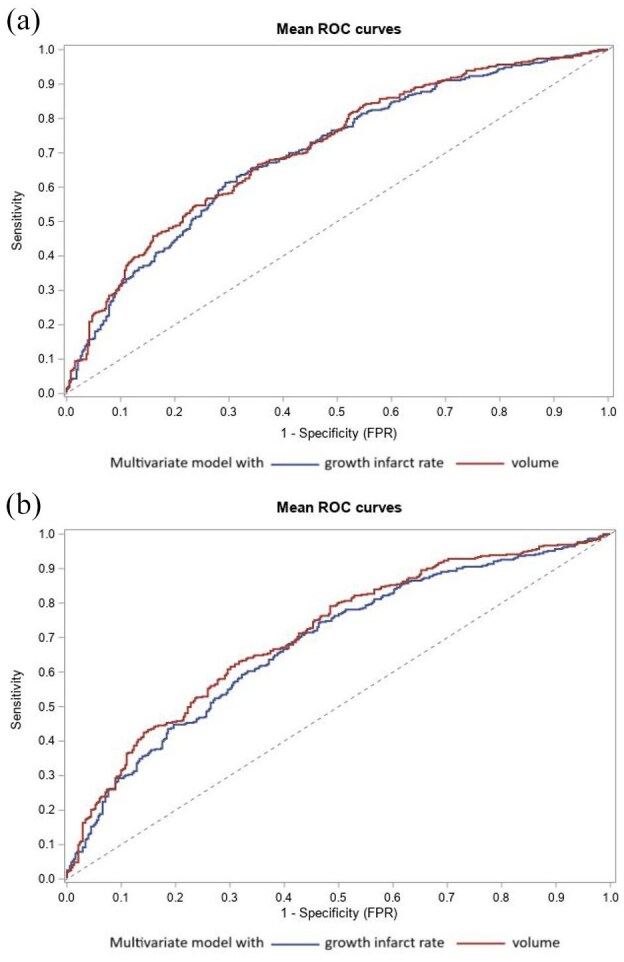
Receiver operating characteristic (ROC) curve of IGR and baseline infarct volume to predict any HT (a) and PH (b).

The multivariate model to predict the occurrence of any HT (including all other independent predictors of HT and IVT; see Supplemental Table 2) had a significantly better AUC with the baseline infarct volume than with IGR (0.717, 95% CI 0.697–0.754 and 0.698, 95% CI 0.659–0.736 respectively, *p*-value = 0.019) (Figure 2). Similarly, the multivariate model to predict PH occurrence (including all other independent predictors of PH and IVT; see Supplemental Table 4) had a significantly better AUC with the baseline infarct volume than with IGR (0.734, 95% CI 0.689–0.778 and 0.704, 95% CI 0.657–0.751 respectively, *p*-value = 0.029).

## Discussion

In this retrospective analysis of the ETIS and FRAME studies, we identified a significant association between a faster IGR and a significantly increased risk of any HT. Despite not reaching significance, we also observed that a faster IGR might also increase the risk of parenchymal hematoma. However, we also found that the predictive strength of IGR was significantly inferior to the predictive value of the initial infarct volume on admission imaging.

These results first suggest a robust link between the IGR and the likelihood of any HT. The non-significance in multivariable analysis observed for parenchymal hematoma also suggests that other factors, such as age, diabetes and baseline NIHSS score, must also be involved in the HT pathophysiology, along with a potential lack of statistical power of our analyses in this less frequent HT subtype.

Though the predictors of HT after stroke have already been questioned through literature, the possible link between the IGR and HT remains under-investigated. In addition to other predictors of HT already identified in the literature, the independent role of IGR deserved specific investigations. This contributes to an individualized assessment of each patient’s hemorrhagic risk, taking into account clinical, biological and radiological features. An indirect concordant signal has already been observed in previous publications reporting increased odds of HT in patients with large ischemic core on baseline imaging and receiving IVT.^17,18^ Indeed, IVT being administered within the usual early time window after onset, the large core was an indirect marker of a fast IGR. In an ancillary analysis of the SWIFT-DIRECT trial, we found that a faster IGR was associated with increased risks of HT.^11^ More, fast progressors experienced an increased risk of HT if treated with IVT at the acute phase. Also, in this recent work, we observed that the IGR was non-linear.^16^ Three stroke progression patterns were defined as slow, intermediate and fast progressors and associated with different likelihoods of HT.^16^ These previous findings along with our presented results tend to demonstrate the influence of IGR on the risk of subsequent HT. Importantly, it can be hypothesized that worse prognosis in fast progressors might not only be mediated by larger and earlier cerebral infarctions but also by an increased risk of HT.

Several hypotheses could explain the mechanisms by which the IGR is associated with the risk of HT. One of the main hypotheses is the more rapid, earlier and more severe alteration of the BBB in cases of rapid progression. Previous studies have reported a significant increase in the risk of HT in the context of BBB alterations.^27,28^ Further research is warranted to explore the underlying mechanisms driving these associations and to refine predictive models for hemorrhagic risks in stroke patients.

Yet, we also observed that the ability to predict the occurrence of HT was significantly better with the baseline infarct volume alone than with IGR that combines volume and time. This result might be explained by the calculation methodology itself for the definition of IGR. Indeed, rather than merging two predictors of HT together into a stronger individualized variable, the baseline infarct volume was actually divided by the time since stroke onset. Time being on the denominator could therefore possibly counterbalance the effect of volume in some cases. Altogether, this approach may have actually reduced the statistical association of the two covariables (volume and time) that were combined to build the IGR. Given its easy measurement and direct perception in daily practice, infarct volume on admission imaging seems more relevant to evaluate the risk of secondary HT and mitigate the use of IGR for HT prediction. Despite the recent growing and still ongoing interest on the value of IGR in the setting of acute ischemic stroke, one can question the added value of this constructed baseline metric for HT risk assessment in comparison with a straightforward data being infarct volume on baseline imaging.^15^

These results also suggest the need for tailored therapeutic strategies in fast progressors. This may include strict blood pressure and glycemic control, but also avoiding anti-thrombotic therapy during the early phase. The development of neuroreparative or neuroprotective treatments targeting the blood brain barrier might be expected.^27^ Novel antiplatelet agents with reduced hemorrhagic risk also appear a promising area of research in these patients.^28^

Several limitations must be acknowledged. First, we focused on patients considered eligible for EVT which may have constituted a selection bias, with patients generally more clinically severe than those with more distal occlusions. The study population might be a reflect only of patients considered for EVT at the time of the study period. Patients with poorer clinical and/or imaging features such as fast or ultrafast progressors were likely often not considered for mechanical thrombectomy and therefore underrepresented in this study. Differences in baseline characteristics between ETIS and FRAME patients may have had influenced our results. Especially, the rates of morthership versus drip-&-ship admissions and the time between imaging and EVT were different in these two cohorts. Secondly, the utilization of MRI limited the generalizability of our findings to CT as the primary imaging modality. However, given the higher sensitivity of DWI in early ischemic changes detection, this approach, together with the inclusion of patients with a known time of symptom onset only, allowed a high accuracy in IGR measurements. Then, in our study, given their well-established value and to avoid multiple statistical analyses, we investigated only imaging definitions of HT. The rate of HT in our cohort aligns with the highest rates reported in the literature. Notably, many studies have relied solely on CT for assessing HT. In contrast, our study used MRI for a relatively large proportion of patients (39%), which has an established greater sensitivity for HT detection.^29^ As a result, our HT rate was slightly higher than in studies using only CT, but it is consistent with the publications in which MRI was used for a large part of the study population.^2,3,5^ Also, variations in the HT assessment between the FRAME and ETIS studies might have marginally influenced the results. Time to recanalization would have been of interest in this study but was not available. Lastly, the study may suffer from a lack of statistical power which likely explained the non-significance of the association between parenchymal hematoma occurrence and IGR, as this HT subtype was less frequent.

## Conclusion

In patients presenting stroke with LVOS intended for EVT, a faster IGR was significantly associated with an increased risk of any HT but not with the occurrence of PH. However, the baseline infarct volume was a stronger predictor of HT than IGR. This finding might mitigate the interest of IGR in providing additional value for the HT risk assessment at the acute phase of a LVOS treated with EVT.

## Supplementary Material

sj-pdf-1-eso_23969873251357151

## Non-Standard Abbreviations

aOR: Adjusted Odds Ratio

ASITN CS: American Society of Interventional and Therapeutic Neuroradiology Collateral Grading System

ASPECTS: Alberta Stroke Program Early Computed Tomography Score

BBB: Blood- Brain Barrier

CI: Confidence interval

DWI: Diffusion Weighted Imaging

ETIS: Endovascular Treatment in Ischemic Stroke

EVT: Endovascular Treatment

FRAME: French Acute Multimodal Imaging Study to Select Patients for Mechanical Thrombectomy

HIR: Hypoperfusion Index Ratio

HT: Hemorrhagic Transformation

IGR: Infarct Growth Rate

IVT: Intravenous Thrombolysis

LVOS: Large Vessel Occlusion Stroke

NIHSS: National Institutes of Health Stroke Scale

OR: Odds Ratio

SD: Standard Deviation
