# Supplementary figures and images for: Hemorrhagic transformation after endovascular treatment: Baseline infarct volume is a better predictor than infarct growth rate
Source: Eur Stroke J. 2026 Jan 1;11(1):23969873251357151. doi: 10.1093/esj/23969873251357151 (PMC12866234; doi:10.1093/esj/23969873251357151)

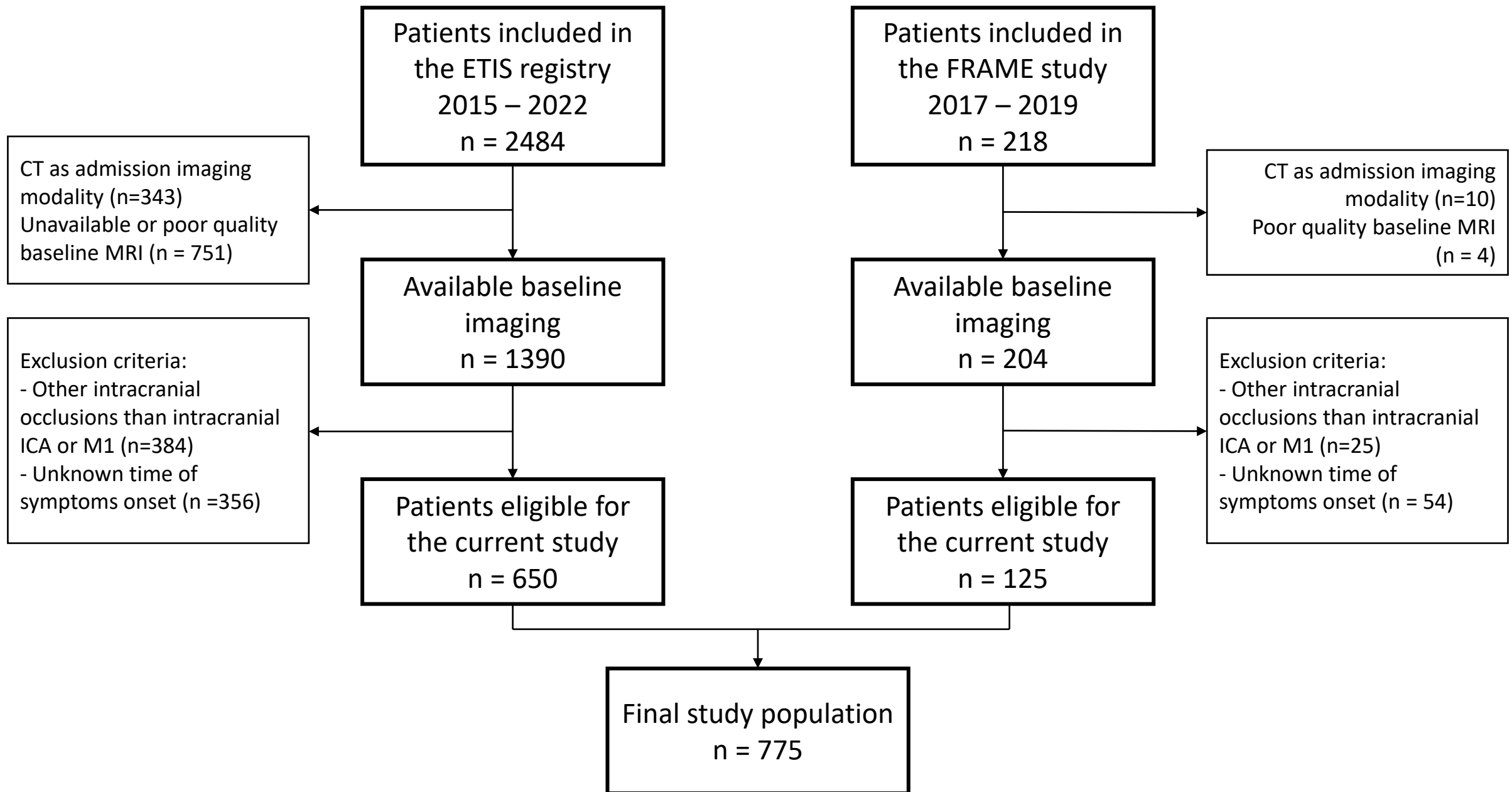

Supplement: sj-pdf-1-eso_23969873251357151 [file sj-pdf-1-eso_23969873251357151.pdf]
